# Author Correction: DNA-guided transcription factor interactions extend human gene regulatory code

**DOI:** 10.1038/s41586-025-09129-1

**Published:** 2025-06-03

**Authors:** Zhiyuan Xie, Ilya Sokolov, Maria Osmala, Xue Yue, Grace Bower, J. Patrick Pett, Yinan Chen, Kai Wang, Ayse Derya Cavga, Alexander Popov, Sarah A. Teichmann, Ekaterina Morgunova, Evgeny Z. Kvon, Yimeng Yin, Jussi Taipale

**Affiliations:** 1 State Key Laboratory of Cardiovascular Diseases and Medical Innovation Center, Shanghai East Hospital, School of Medicine, Tongji University, Shanghai, China; 2 Department of Biochemistry, University of Cambridge, Cambridge, UK; 3 Generative and Synthetic Genomics Programme, Wellcome Sanger Institute, Hinxton, UK; 4 Applied Tumor Genomics Program, Biomedicum, University of Helsinki, Helsinki, Finland; 5 Department of Developmental and Cell Biology, University of California, Irvine, Irvine, CA USA; 6 Cellular Genetics Programme, Wellcome Sanger Institute, Hinxton, UK; 7 European Synchrotron Radiation Facility (ESRF), Grenoble, France; 8 Department of Medicine and Cambridge Stem Cell Institute, University of Cambridge, Cambridge, UK; 9 Department of Medical Biochemistry and Biophysics, Karolinska Institutet, Stockholm, Sweden; 10 Clinical Center for Brain and Spinal Cord Research, Tongji University, Shanghai, China

**Keywords:** Transcriptional regulatory elements, High-throughput screening, Systems analysis

Correction to: *Nature* 10.1038/s41586-025-08844-z Published online 9 April 2025

In the version of the article initially published, in Extended Data Fig. 7a, the image for “#3 (Tandem)” was a duplicate of “#2 (Tandem)”. Extended Data Fig. 7a has now been amended with the correct “#3 (Tandem)” image in the HTML and PDF version of the article.

