# Supplementary material for: DNA-guided transcription factor interactions extend human gene regulatory code
Source: Nature. 2025 Apr 9;641(8065):1329–38. doi: 10.1038/s41586-025-08844-z (PMC12119339; doi:10.1038/s41586-025-08844-z)
Supplement: Supplementary file 1 — Reporting Summary [file 41586_2025_8844_MOESM1_ESM.pdf]

nature portfolio

Corresponding author(s): Yimeng Yin & Jussi Taipale

Last updated by author(s): Feb 10, 2025

Reporting Summary

Nature Portfolio wishes to improve the reproducibility of the work that we publish. This form provides structure for consistency and transparency in reporting. For further information on Nature Portfolio policies, see our Editorial Policies and the Editorial Policy Checklist.

Statistics

For all statistical analyses, confirm that the following items are present in the figure legend, table legend, main text, or Methods section.

n/a	Confirmed	
☐	☒	The exact sample size (*n*) for each experimental group/condition, given as a discrete number and unit of measurement
☒	☐	A statement on whether measurements were taken from distinct samples or whether the same sample was measured repeatedly
☐	☒	The statistical test(s) used AND whether they are one- or two-sided<br>*Only common tests should be described solely by name; describe more complex techniques in the Methods section.*
☒	☐	A description of all covariates tested
☐	☒	A description of any assumptions or corrections, such as tests of normality and adjustment for multiple comparisons
☐	☒	A full description of the statistical parameters including central tendency (e.g. means) or other basic estimates (e.g. regression coefficient) AND variation (e.g. standard deviation) or associated estimates of uncertainty (e.g. confidence intervals)
☐	☒	For null hypothesis testing, the test statistic (e.g. *F*, *t*, *r*) with confidence intervals, effect sizes, degrees of freedom and *P* value noted<br>*Give P values as exact values whenever suitable.*
☒	☐	For Bayesian analysis, information on the choice of priors and Markov chain Monte Carlo settings
☒	☐	For hierarchical and complex designs, identification of the appropriate level for tests and full reporting of outcomes
☒	☐	Estimates of effect sizes (e.g. Cohen's *d*, Pearson's *r*), indicating how they were calculated

Our web collection on statistics for biologists contains articles on many of the points above.

Software and code

Policy information about availability of computer code

Data collection	Crystallographic data were collected using the software developed in synchrotron beam-line ID23-1 in ESRF and listed in Material and Method section
Data analysis	MOtifSTAtistic Software Suite v1.1 (MOSTA-SSTAT); tomtom from meme 5.4.1; motifStack v1.38.0; circos v0.69-8; cytoscape v3.10.1; gapped k-mer similarity (https://github.com/jutaipal/motifsimilarity); moods-1.9.4.1; perl v5.34.1; GLPSOL--GLPK LP/MIP Solver 5.0; R 4.3.0 and the compatible packages; MoSBAT-AffiMx v9.0 (https://github.com/csglab/MoSBAT); Crystallographic data analysis: XDS and CCP4 suits 7.1 and 8.0; MR and refinement: Phaser and Refmac5 as implemented in CCP4 and Phenix.refine; Model building: Coot (versions 0.9.6 and o.9.8.92 (EL)) as implemented in CCP4 and Phenix; Structural visualisation: PyMol 2.5.4. custom code links: https://github.com/YinLabTJ/Relative_Affinity_CAP-SELEX; https://github.com/YinLabTJ/MI_CAP-SELEX; https://github.com/MariaOsmala/TFBS; https://github.com/MariaOsmala/TFBS-evolutionary-conservation; https://github.com/i-l-sokolov/RosettaFoldNA_output_analysis.

For manuscripts utilizing custom algorithms or software that are central to the research but not yet described in published literature, software must be made available to editors and reviewers. We strongly encourage code deposition in a community repository (e.g. GitHub). See the Nature Portfolio guidelines for submitting code & software for further information.

Data

Policy information about availability of data

All manuscripts must include a data availability statement. This statement should provide the following information, where applicable:

- Accession codes, unique identifiers, or web links for publicly available datasets
- A description of any restrictions on data availability
- For clinical datasets or third party data, please ensure that the statement adheres to our policy

All sequence data are available in ENA, under accession number PRJEB66722. Structure coordinates are available in PDB (entries: 8R7Z, 8R7F, 8BZM, 8BYX, 5NO6, and 5EG0). The ChIP-seq data used in this study are from Encyclopedia of DNA Elements (ENCODE) with accession number ENCSR786QMI, ENCSR918LYT, ENCFF517TKD, ENCFF766VUQ, ENCFF641ZFM, ENCFF992LDJ, ENCFF882AEU, ENCFF093KLR, ENCFF053UZX, ENCFF146SYU, ENCFF164MPE, ENCFF294IGP, ENCFF601WHE, ENCFF451AII, ENCFF934JFA, ENCFF637UJN, ENCFF170IZO, ENCFF172KBM, ENCFF118UKC, ENCFF827VVQ, ENCFF700TAS, ENCFF114CWH, ENCFF440FTA, ENCFF308NZT, ENCFF100KKH, ENCFF885PQR, ENCFF519GKM, ENCFF929XBL, ENCFF839JEO, ENCFF890WJZ, ENCFF788ONH, ENCFF398TIE, ENCFF712HHN, ENCFF314KET, ENCFF399YOO, ENCFF703JHN, ENCFF023EDF, ENCFF488DML, ENCFF603PUS, ENCFF164JZB, ENCFF355HRT, ENCFF605GXY, ENCFF331BSI, ENCFF132AJP, ENCFF314CDT, ENCFF919ZSN, ENCFF011QFM, ENCFF696OTJ, ENCFF358INM, ENCFF759YCY, ENCFF798IVN, ENCFF005YUC, ENCFF647OBG, ENCFF904DOZ, ENCFF634YFK, ENCFF943XDP, ENCFF438PCR, ENCFF718VHT, ENCFF489EME, ENCFF250MUC, ENCFF951BFN, ENCFF493DXA, ENCFF882ARK, ENCFF065RZP, ENCFF970QKS, ENCFF763MXW, ENCFF715WGN, ENCFF118GMS, ENCFF324ELP, ENCFF518EGY, ENCFF566PRZ, ENCFF438IYI, ENCFF592NJN, ENCFF289ZIR, ENCFF594YBN, ENCFF112JVK, ENCFF030DLI, ENCFF091NQE, ENCFF427XNV, ENCFF751VAZ, ENCFF131TYZ, ENCFF987QLI, ENCFF381ULU, ENCFF290IBP, ENCFF463DWW, ENCFF567HPV, ENCFF833ZAC, ENCFF946KCK, ENCFF219OPP (See also Supplementary Table S2). Genome assembly GRCh38 is downloaded from UCSC (ftp://hgdownload.soe.ucsc.edu/apache/htdocs/goldenPath/hg38/bigZips/hg38.fa.masked.gz).

Research involving human participants, their data, or biological material

Policy information about studies with human participants or human data. See also policy information about sex, gender (identity/presentation), and sexual orientation and race, ethnicity and racism.

Reporting on sex and gender	n/a
Reporting on race, ethnicity, or other socially relevant groupings	n/a
Population characteristics	n/a
Recruitment	n/a
Ethics oversight	n/a

Note that full information on the approval of the study protocol must also be provided in the manuscript.

Field-specific reporting

Please select the one below that is the best fit for your research. If you are not sure, read the appropriate sections before making your selection.

☒ Life sciences ☐ Behavioural & social sciences ☐ Ecological, evolutionary & environmental sciences

For a reference copy of the document with all sections, see nature.com/documents/nr-reporting-summary-flat.pdf

Life sciences study design

All studies must disclose on these points even when the disclosure is negative.

Sample size	We haven't formally calculated the sample sizes required. However, the sequencing depth of CAP-SELEX libraries was set to ensure that at least hundreds of thousands unique reads are available for each TF. Under this sample size, if a TF is binding DNA without restrictions, any non-random pattern of TF binding that has a biologically meaningful effect size (as observed in our study) can only occur with an extremely small p-value.
Data exclusions	The failed CAP-SELEX experiments were excluded according to the QC criteria. The criteria define successful CAP-SELEX experiments as having enriched motifs for both TF1 and TF2. The exclusion criteria is established before we perform conclusion-related analyses.
Replication	We performed multiple cycles (3) of CAP-SELEX for each TF pair. Each cycle is essentially a replicate of the same experiment. In addition, the whole CAP-SELEX procedure was also repeated for all TFs pairs. For all the reported signals, their enrichment is observed across multiple SELEX cycles, and are reproducible between two or more independent batches of SELEX.
Randomization	No grouping was involved in the experiments - no randomization was conducted as a result.
Blinding	Most analyses were performed using computational algorithms. Investigators were not blinded

Reporting for specific materials, systems and methods

We require information from authors about some types of materials, experimental systems and methods used in many studies. Here, indicate whether each material, system or method listed is relevant to your study. If you are not sure if a list item applies to your research, read the appropriate section before selecting a response.

Materials & experimental systems

n/a	Involved in the study
☒	☐ Antibodies
☒	☐ Eukaryotic cell lines
☒	☐ Palaeontology and archaeology
☐	☒ Animals and other organisms
☒	☐ Clinical data
☒	☐ Dual use research of concern
☒	☐ Plants

Methods

n/a	Involved in the study
☒	☐ ChIP-seq
☒	☐ Flow cytometry
☒	☐ MRI-based neuroimaging

Animals and other research organisms

Policy information about studies involving animals; ARRIVE guidelines recommended for reporting animal research, and Sex and Gender in Research

Laboratory animals	All animals used in this study were of Mus musculus species and FVB/NCrl strain at ages E11.5
Wild animals	Study did not use wild animals.
Reporting on sex	Gender was not identified during collections; however, it is assumed that the groups contained approximately equal numbers of male and female mice
Field-collected samples	Study did not use field-collected samples.
Ethics oversight	This research complies with all relevant ethical regulations. All animal procedures, including those related to the generation of transgenic animals, were conducted in accordance with the guidelines of the National Institutes of Health (NIH) and approved by the Institutional Animal Care and Use Committee at the University of California, Irvine under protocol no. AUP-23-005.

Note that full information on the approval of the study protocol must also be provided in the manuscript.

Plants

Seed stocks	n/a
Novel plant genotypes	n/a
Authentication	n/a
